# Targeted metabolomic profiles of serum amino acids and acylcarnitines related to gastric cancer

**DOI:** 10.7717/peerj.14115

**Published:** 2022-10-06

**Authors:** Dehong Li, Yan Lu, Fenghui Zhao, Li Yan, Xingwen Yang, Lianhua Wei, Xiaoyan Yang, Xiumei Yuan, Kehu Yang

**Affiliations:** 1 Evidence Based Medicine Center, School of Basic Medical Sciences, Lanzhou University, Lanzhou, China; 2 Department of Clinical laboratory, Gansu Provincial Hospital, Lanzhou, China; 3 Department of Pathology, Gansu Provincial Hospital, Lanzhou, China

**Keywords:** Gastric cancer, Atrophic Gastritis, Superficial gastritis, Amino acids, Acylcarnitines, Metabolomics

## Abstract

**Background:**

Early diagnosis and treatment are imperative for improving survival in gastric cancer (GC). This work aimed to assess the ability of human serum amino acid and acylcarnitine profiles in distinguishing GC cases from atrophic gastritis (AG) and control superficial gastritis (SG) patients.

**Methods:**

Sixty-nine GC, seventy-four AG and seventy-two SG control patients treated from May 2018 to May 2019 in Gansu Provincial Hospitalwere included. The levels of 42 serum metabolites in the GC, AG and SG groups were detected by liquid chromatography-tandem mass spectrometry (LC–MS/MS). Then, orthogonal partial least squares discriminant analysis (OPLS-DA) and the Kruskal-Wallis H test were used to identify a metabolomic signature among the three groups. Metabolites with highest significance were examined for further validation. Receiver operating characteristic (ROC) curve analysis was carried out for evaluating diagnostic utility.

**Results:**

The metabolomic analysis found adipylcarnitine (C6DC), 3-hydroxy-hexadecanoylcarnitine (C16OH), hexanoylcarnitine (C6), free carnitine (C0) and arginine (ARG) were differentially expressed (all VIP >1) and could distinguish GC patients from AG and SG cases. In comparison with the AG and SG groups, GC cases had significantly higher C6DC, C16OH, C6, C0 and ARG amounts. Jointly quantitating these five metabolites had specificity and sensitivity in GC diagnosis of 98.55% and 99.32%, respectively, with an area under the ROC curve (AUC) of 0.9977.

**Conclusion:**

This study indicates C6DC, C16OH, C6, C0 and ARG could effectively differentiate GC cases from AG and SG patients, and may jointly serve as a valuable circulating multi-marker panel for GC detection.

## Introduction

Gastric cancer (GC) represents a malignancy originating from the epithelium of the gastricmucosa (Lai, 2019). It ranks fifth and third among cancers in terms of incidence and mortality, respectively (Bray et al., 2018). In China, an estimated 679,100 incident GC cases are detected yearly, and GC represents the second deadliest malignancy in both men and women (Chen et al., 2016). Gansu province, located in the Northwest of China, represents an area with high GC incidence and mortality (Guo et al., 2020). As such, further studies of GC in this area are warranted. The majority of early GC cases show no overt symptoms. In some cases, nausea, vomiting, and/or other upper gastrointestinal tract symptoms are observed, which are not specific (Gong & Zhang, 2020). Most GC cases are diagnosed at an advanced stage, owing to the disease being mostly asymptomatic in the early stage as well as the unavailability of adequate screening methods. At present, GC treatment mainly relies on surgery, in combination with radiation therapy and chemotherapy (He et al., 2020). Surgery followed by chemotherapy in GC increases survival in the early stage, but not in the intermediate and advanced stages (Zhou et al., 2020).

Early detection and timely treatment following preciserisk classification are imperative for improving patients outcome in GC. Endoscopic assessment represents a very reliable method, which could perform GC diagnosis but is expensive and carries inherent risks related to all invasive procedures, limiting its clinical applicability (Chan et al., 2014). Conventional serum tumor biomarkers have been proposed as useful parameters for early detection, prognostic prediction and recurrence monitoring in GC (Emoto et al., 2012). Nonetheless, most circulating molecular markers are not recommended for early GC diagnosis, with limited specificity and sensitivity (Wu et al., 2019). Cell-free nucleic acids (cfNAs) are potential biomarkers for early detection and monitoring of GC (Wan & Zhang, 2016). However, their heterogeneous origin differences in specimen collection and storage, diverse assessment techniques; and various analytical methods may all affect the levels of cfNAs to limit their use as biomarkers (He et al., 2015). The identification and validation of GC-specific biomarkers are therefore warranted to facilitate early diagnosis.

GC has a multifactorial etiology, which is influenced by several genetic, environmental and predisposing factors (Agkoc et al., 2010). It is characterized by a stepwise progression from no active gastritis to chronic active gastritis, precursor GC lesions (atrophy, intestinal metaplasia and dysplasia), and finally gastric adenocarcinoma (Kuligowski et al., 2016). Atrophic gastritis (AG) is a precancerou scondition, with elevated risk of developing gastricneoplasias, including intestinal adenocarcinomas and gastric neuroendocrine tumors (Annibale, Esposito & Lahner, 2020). Therefore, it is necessary to observe the progression from gastritis to gastric cancer, and identifying diagnosis biomarkers for GC is of great significance.

Metabolomic analysis is a promising approach that provides opportunities to elucidate complex tumor-associated metabolic changes and accelerates the process of identifying new tumor biomarkers (Aboud & Weiss, 2013). Metabolic ailments are critical in carcinogenesis and cancer progression, and considered important hallmarks of cancer (Lario et al., 2017). Metabolomic profiling might help determine cancer markers, providing clues for early cancer diagnosis. Metabolomics is considered a rapid and efficient tool for identifying new cancer biomarkers, and serves as a complementary method to gene and proteome analyses (Corona et al., 2012). The serum is commonly considered a pool of metabolites (López-Bascón et al., 2016) and reflects the systemic metabolic regulation in cancer patients. Multiple methods have been developed for metabolome assessment, including nuclear magnetic resonance spectroscopy (NMR), mass spectroscopy (MS), and liquid (LC) and gas (GC) chromatography (Chan et al., 2014). To date, LC-tandem mass spectroscopy (LC-MS/MS)-based high throughput techniques are broadly utilized, allowing joint assessment of multiple metabolites, including amino acids and acylcarnitines, only requiring small amounts of biological specimens (Sarker et al., 2019).

Dysregulated metabolism of glucose, amino acids, lipids, and nucleotides has been demonstrated during gastric carcinogenesis (Huang et al., 2020). It is currently admitted amino acids, in lieu of glucose, are the main drivers of carbon-based biomass production in fast-growing malignant cells. In addition, amino acids have nitrogen, representing the major nitrogen source for hexosamines, nucleotides and other nitrogenous molecules in fast-growing cells (Choi & Coloff, 2019). Acylcarnitines constitute obligate cofactors in mitochondrial fatty acid *β*-oxidation, which represents the major step of energy production and is altered in multiple malignancies (Lu et al., 2019). Excessive acylcarnitine production might reflect altered fatty acid oxidation, in turn contributing to metabolic diseases. In cancer, acylcarnitine metabolism is considered a gridlock to precisely promote metabolic flexibility based on its crucial function in controlling the metabolic processes of glucose and fatty acids. Metabolic reprogramming in malignant cells modulates the production of acylcarnitines of different chain lengths. This intercalates acylcarnitines with other major metabolic pathways, factors and metabolites, resulting in balanced energy production and consumption, as well as in the biosynthesis of metabolic intermediates for fast growth (Li, Gao & Jiang, 2019). Amino acids and acylcarnitines are potential biomarkers for cancer diagnosis, with critical roles in cell physiology as baseline metabolites and metabolic modulators. However, studies evaluating amino acid and acylcarnitine profiles in association with gastric cancer are scarce and inconsistent. Jing et al. (2018) compared 22 plasma amino acids of gastric ulcer and gastric cancer patients, and demonstrated four amino acids (glutamine, histidine, arginine and tryptophan) show reduced amounts, whereas ornithine content was increased in plasma specimens from patients with GC. Miyagi et al. (2011) observed the difference of 19 plasma amino acids levels between normal people and gastric cancer patients and found that threonine, glutamine, alanine, citrulline, leucine, phenylalanine, lysine, asparagine, methionine, citrulline, valine, tryptophan, histidine and arginine were decreased in cancer. However, another study found that plasma arginine level in the gastric cancer group was significantly higher than that in the gastric ulcer, gastric polyp, and gastritis groups (Shi et al., 2021). A similar situation was found for acylcarnitines, Corona et al. (2018) found that serum acetylcarnitine (C2), hexadecanoylcarnitine (C16) and octadecenoylcarnitine (C18:1) of GC patients were higher than first-degree relatives. A study performed by Lario et al. (2017) found that plasma hydroxytetradecadienylcarnitine (C14:2-OH) and octadecanoylcarnitine (C18) were increased in GC patients compared to non-active gastritis (NAG), chronic active gastritis (CAG) and precursor lesions of gastric cancer (PLGC). Therefore, more studies are required to assess metabolic changes in gastric cancer.

The present work primarily aimed to observe changes in serum metabolomics during the whole process from superficial gastritis (SG) to AG eventually progressing to GC, and to identify a potential biomarker panel to distinguish GC from SG and AG.

## Materials & Methods

### Patients

A total of 215 participants treated in Gansu Provincial Hospital between May 2018 and May 2019 were enrolled, including 69 GC *patients in General Surgery Wards 1 and 2, 74 AG patients* in the Department of Gastroenterology, and 72 SG patients undergoing routine physical examination (controls).

Inclusion criteria for GC cases were: (1) meeting the diagnostic guidelines of the CSCO gastric cancer expert committee (Wang et al., 2019); (2) new diagnosis of primary GC without other cancers; (3) no previous GC-related reatment, chemotherapy or surgical treatment. Exclusion criteria were: (1) tumor recurrence; (2) pregnancy. The inclusion criterion for AG cases was a diagnosis by gastroscopy and pathological examination. A gastrointestinal pathologist diagnosed and evaluated pathological specimens for the degree of gastricatrophy according to USS (Dixon et al., 1996). The 72 patients with SG were subjected to endoscopic diagnosis. Exclusion criteria were SG with erosion, bleeding, and bile reflux. The present trial had approval from the Institutional Review Board of Gansu Provincial Hospital (2020-173), and all patients provided written informed consent.

### Specimen collection and preparation

Blood samples were obtained from the antecubital vein (approximately 4 mL) in all participants after 12 h of fasting. The collected blood was separated by centrifugation at 3000 rpm for 10 min (KDC-2046; Anhui USTC Zonkia Scientific Instrument Co., Ltd., Anhui, China), with in 1 h after collection. The obtained serum was used to prepare dried serum spot (DSS) specimens utilizing dry blood filter paper, followed by immediate storage at −80 °C until use.

Before experiment start, DSS papers were punched into 3.2-mm diameter discs, which were placed in 96-well microplates (Perkin Elmer Life Sciences, WallacOy, Turku, Finland) for the extraction of amino acids and acylcarnitines. Two high-and low-level quality controls (alanine, citrulline, glycine, leucine, methionine, phenylalanine, proline, tyrosine, valine, free carnitine, acetylcarnitine, propionylcarnitine, butyrylcarnitine, isovalerylcarnitine, glutarylcarnitine, hexanoylcarnitine, octanoylcarnitine, decanoylcarnitine, dodecanoylcarnitine, tetradecanoylcarnitine, hexadecanoylcarnitine and octadecanoylcarnitine) were individually added to the third, fourth, and the last two wells of each plate. The first and second wells were used as blanks. Each well was added 100 µL of the working solution (internal standard prepared with the extraction solution according to the kit’s instructions). After sealing, the plates were shaken at 700 rpm for 45 min at 45 °C. Following incubation, 75 µL filtrate was collected in new 96-well plates for LC-MS/MS.

### Reagents and Chemicals

High-purity water was obtained from Synergy (MILLIPORE), and methanol was purchased from Thermo Fisher Chemicals (Waltham, MA, USA). Isotope-labeled internal standards and acylcarnitine isotope-labeled internal standards for absolute quantification were provided by PerkinElmer (PerkinElmer Life Sciences, Waltham, MA, USA). The extraction and flow solutions were provided by PerkinElmer.

### LC-MS/MS analysis of small molecule metabolites

A high-performance liquid chromatography detector LC-20AD (Shimadzu,Tokyo, Japan) and a MS/MS detection system AB Sciex 3200 Qtrap (AB Sciex, Framingham, MA, USA) were used to carry out LC-MS/MS. Analyst v1.6.2 (AB Sciex) was utilized for data collection as well as system control. Data were pre-processed with ChemoView2.0.3 (AB Sciex). Scanning was performed in the positive mode. In brief, 20 µL specimen was injected per run at 0.175 mL/min initially, reduced to 0.013 mL/min with in 0.02 min and maintained for 0.8 min. The flow rate was then adjusted to 0.6 mL/min with in 0.01 min, and subsequently kept constant until 0.5min (eluent: methanol, water and oxalic acid). The curtain gas pressure was 25 psi, for an ion spray voltage of 4.5 kV. The ion source gas 1and 2 pressure was 25 psi, and the auxiliary gas was kept at 350 °C.

### Statistical analysis

LC-MS/MS-based metabolomic data for amino acids and acylcarnitine isotopes were submitted to multi variable analysis with SIMCAv14.1 (MKSUMETRICSAB). Orthogonal partial least squares discriminant analysis (OPLS-DA) was carried out for verifying the discrimination of metabolite profiles among the GC, AG and SG groups. A permutation test was carried out to assess the model’s reliability; Variable importance inprojection (VIP) values>1 were selected and used for further univariate analysis. SPSS version19 (SPSS, Inc., USA) was used for univariate analysis. Continuous data were assessed for normality by the Shapiro–Wilktest, and expressed as mean ± SD and median (interquartile range), in case of normal and skewed distributions, respectively. The Kruskal-Wallis and Mann–Whitney U tests were carried out for non-normally distributed variables. GraphPad Prism version 6.04 was used for generating Boxplots. A GC diagnosis model including select metabolites was built by binary logistic regression analysis. The diagnostic value of the obtained regression model was assessed using a receiver operating characteristic (ROC) curve.

## Results

### Clinical characteristics

The clinical characteristics are summarized in Table 1. Serum samples were obtained from 69 patients with GC, 74 with AG, and 72 with SG. The average age of the GC cases was 62 (14) years, which was markedly elevated compared with those of AG(50 (16.25) years, *P* = 0.000) and SG (49 (29) years, *P* = 0.000; no difference was observed in age between the AG and SG patient groups (*P* = 0.735). The male-to-female ratios in the GC and AG groups were starkly elevated compared with that of the SG group (*P* = 0.000 and *P* = 0.000, respectively); no difference was observed between the GC and AG groups (*P* = 0.387).

Among the 69 GC patients, 31 (44.93%) were positive for *H.pylori*, a rate that was markedly elevated compared with that of SG cases (20 positive and 52 negative, *P* = 0.037); no difference was found in the ratio of *H.Pylori* positivity between the GC and AG groups (*P* = 0.616).

There were 9/69 (13.0%) stage I, 37/69 (53.6%) stage II, 20/69 (29.0%) stage III, and 3/69 (4.35%) stage IV GC cases. According to tumor location, 32 individuals had the gastric antrum affected, 24 had the gastric body affected, six had the whole stomach affected, and seven had the fundus-cardia affected. According to the degree of tumor differentiation, there were 39 poorly-differentiated, 23 moderately-differentiated and seven highly-differentiated cases.

### Serum metabolomic profiles of the GC, AG and SG groups

**Figure 1:**
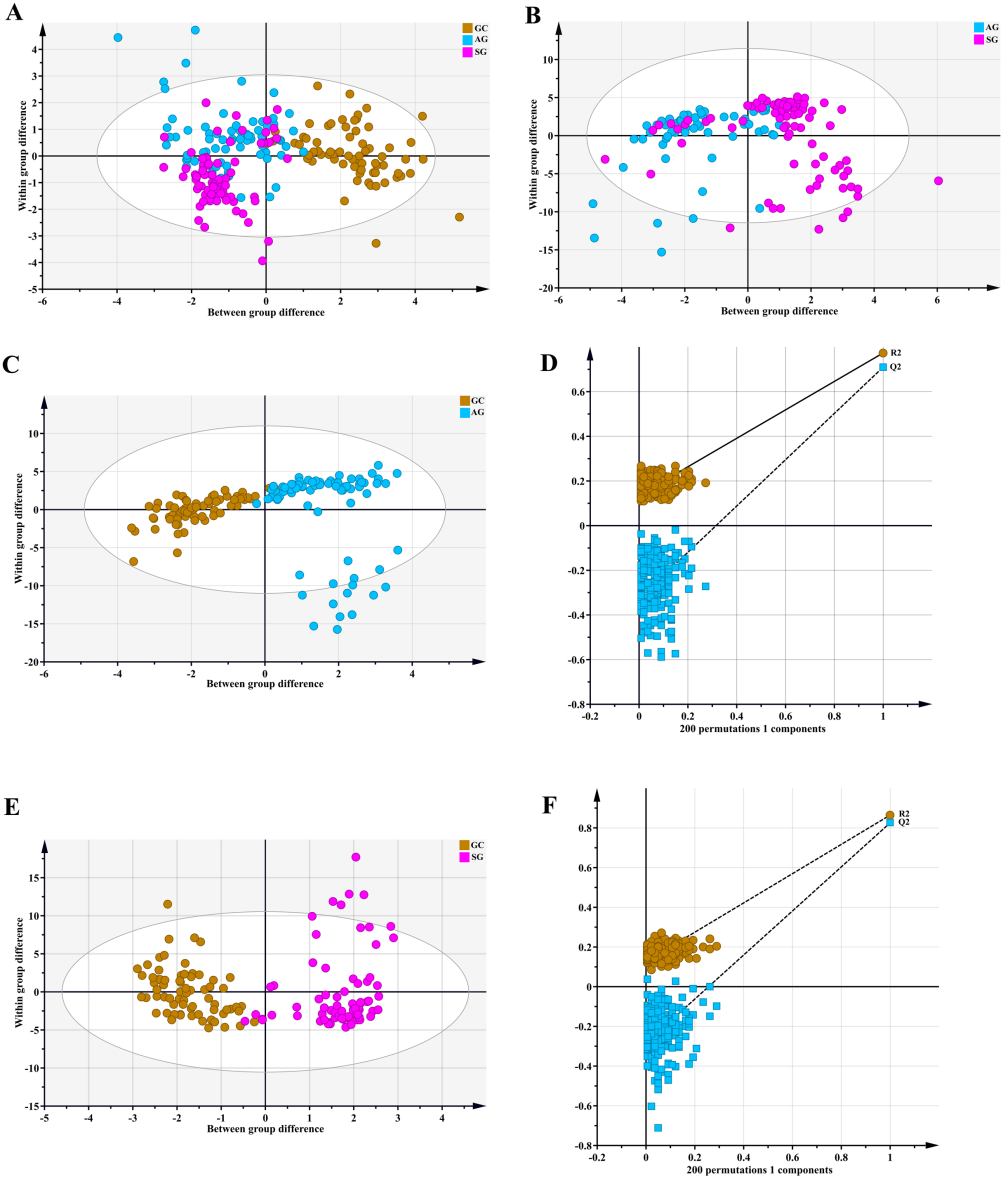
An orthogonal partial least square discriminant analysis (OPLS-DA) model. (A) GC cases tended to cluster to the right, while the AG and SG groups clustered to the left. The OPLS-DA model revealed fairly separated GC from AG and SG. There was no separation between AG and SG. (B) Score plots for AG and SG cases. The model was unable to distinguish AG and SG. (C) and (E) OPLS-DA achieved a fairly distinct separation between GC and AG and between GC and SG. (D) and (F) The 200-time permutation test revealed no overt model over fitting. The *y*-axis intercepts were R2 (0.0, 0.136; 0.0, 0.127) and Q2 (0.0, −0.327; 0.0, −0.284).

Metabolomic profiling utilized the OPLS-DA model that explains the maximum separation among specimens from SG, AG and GC patients. Data are depicted in Fig. 1, with distinct points corresponding to the metabolomic profiles of individual patients. The GC and A G (R2Ycum = 0.773, Q2cum = 0.71) groups (Fig. 1C) and the GC and SG (R2Ycum = 0.864, Q2cum = 0.826) groups (Fig. 1E) were overtly separated. A chance 200-time permutation test revealed R2 and Q2 intercepts of 0.136 and −0.327, and 0.127 and − 0.284, respectively (Figs. 1D and 1F), indicating no model over fitting. However, the OPLS-DA model failed to discriminate between AG and SG groups, as shown in Fig. 1B (R2Ycum = 0.375, Q2cum = 0.293).

### Most significant metabolites

**Figure 2:**
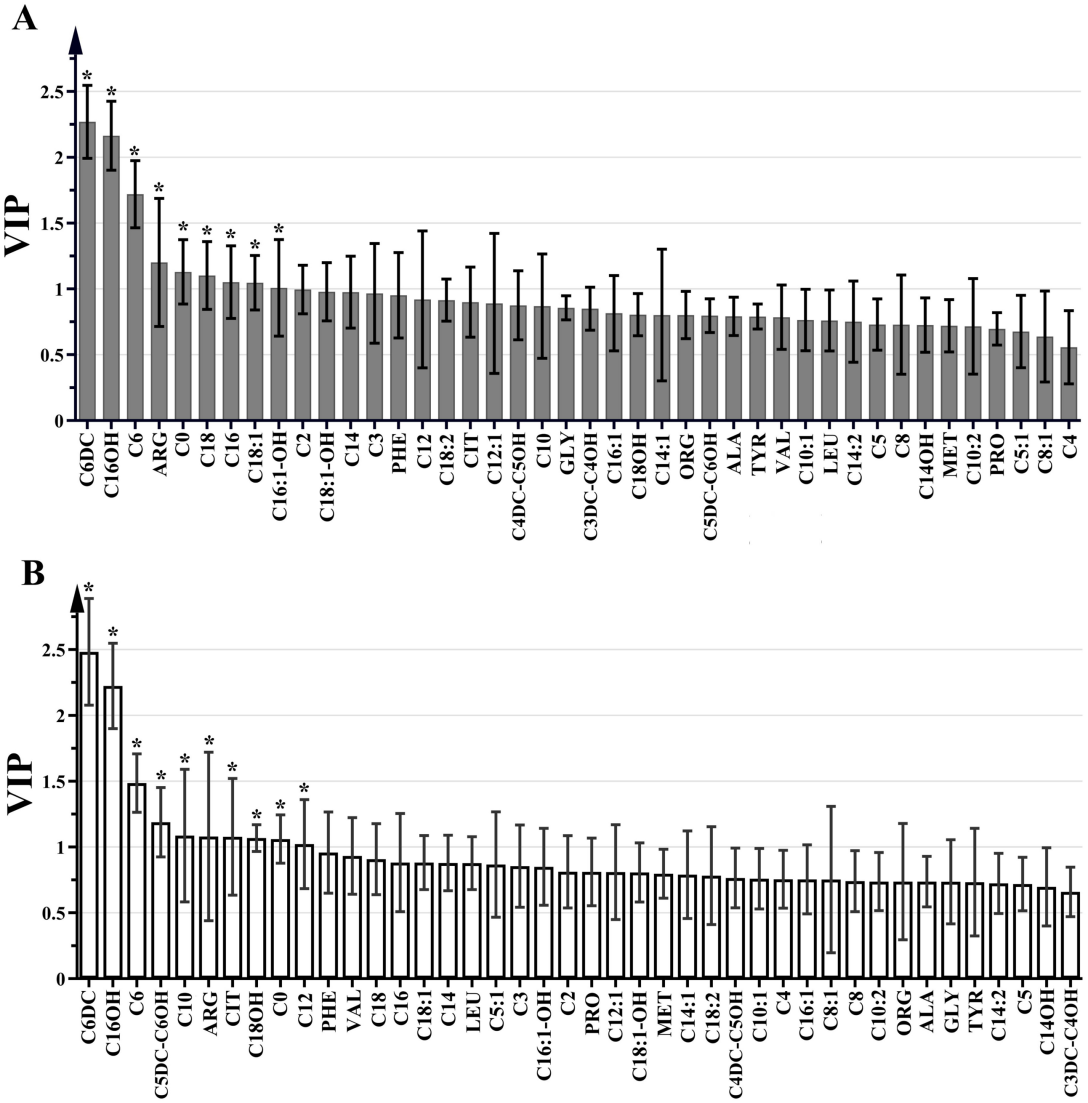
VIP plot for the OPLS-DA model in preselecting variables. (A) GC group *versus* AG group, *VIP >1. (B) GC group *versus* SG group, *VIP >1.

A total of 11 amino acids (alanine, arginine, citrulline, glycine, leucine, methionine, ornithine, phenylalanine, proline, tyrosine and valine) and 31acylcarnitines (free carnitine (C0)), acetylcarnitine (C2), propionylcarnitine (C3), malonylcarnitine/3-hydroxy-butyrylcarnitine (C3DC/C4OH), butyrylcarnitine (C4), methylmalonyl/3-hydroxy-isovalerylcarnitine (C4DC/C5OH), isovalerylcarnitine (C5), tiglylcarnitine (C5:1), glutarylcarnitine/3-hydroxy-hexanoylcarnitine (C5DC/C6OH), hexanoylcarnitine (C6), adipylcarnitine (C6DC), octanoylcarnitine (C8), octenoylcarnitine (C8:1), decanoylcarnitine (C10), decanoylcarnitine (C10:1), decadienoylcarnitine (C10:2), dodecanoylcarnitine (C12), dodecanoylcarnitine (C12:1), tetradecanoylcarnitine (C14), tetradecenoyl-carnitine (C14:1), 3-hydroxy-tetradecanoylcarnitine (C14OH), tetradecadienoylcarnitine (C14:2), hexadecanoylcarnitine (C16), 3-hydroxy-hexadecanoylcarnitine (C16OH), hexadecenoylcarnitin (C16:1), 3-hydroxy-hexadecenoylcarnitine (C16:1OH), octadecanoylcarnitine (C18), 3-hydroxy-octadecanoylcarnitine (C18OH), octadecenoylcarnitine (C18:1), 3-hydroxy-octadecenoylcarnitine (C18:1OH) and octadecadienoylcarnitine (C18:2)) were assessed in each sample of the GC, AG and SG groups. VIP values for pre-selected parameters was utilized to determine indexes responsible for the significant discrimination of the OPLS-DA model. Parameters with VIP>1 were considered to contribute the most to the discrimination of metabolite profiles between the GC and AG groups and between the GC and SG groups (Fig. 2). Significant differences were detected in C6DC, C16OH, C6, ARG, C0, C18, C16, C18:1 and C16:1OH levels between the AG and GC groups. In comparison with the SG group, the GC group had different levels of C6DC, C16OH, C6, C5DC/C6OH, C10, ARG, citrulline, C18OH, C0, and C12. C6DC, C16OH, C6, C0, and ARG were common indicators in the GC group, compared with the SG and AG groups.These results are summarized in Fig. 2.

### Verification of the Most Significant Metabolites

**Figure 3:**
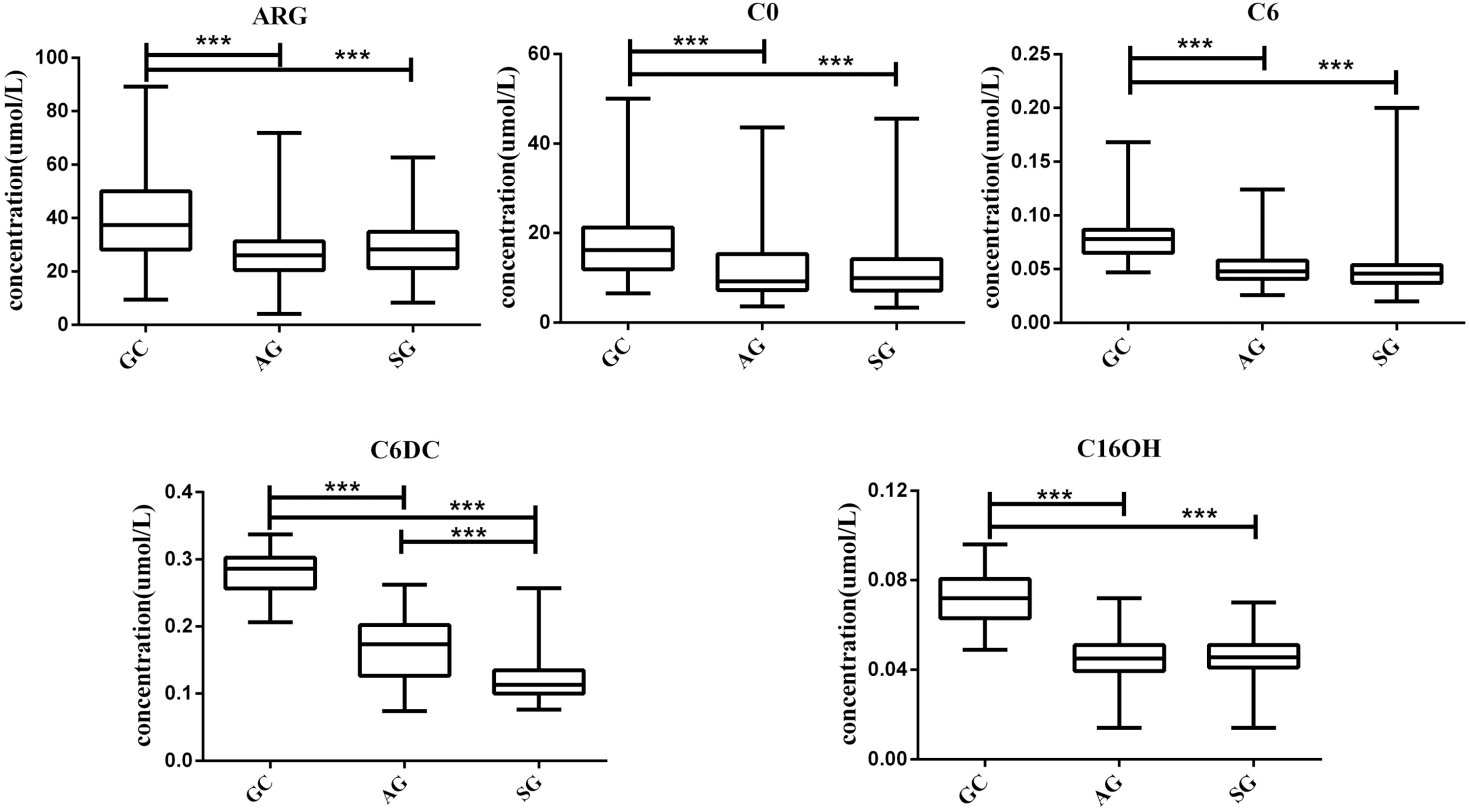
Box plots for select metabolites (Median (interquartile range)) differentiating GC, AG and SG. * *P* < 0.05, ** *p* < 0.001, and *** *p* < 0.0001.

To verify the five metabolites with significant differences, the Kruskal-Wallis H test was performed for multiple-group comparisons, with subsequent Mann–Whitney U-test for group-pair comparisons (Fig. 3). C6DC, C16OH, C6, C0 and ARG were significantly higher in the GC group compared with the AG and SG groups. Meanwhile, C16OH, C6, C0 and ARG showed no differences between the AG and SG groups. These results are summarized in Fig. 3.

### Model performance for metabolites

A binary logistic regression analysis model was built including C6DC, C16OH, C6, C0 and ARG to identify their potential diagnostic values in GC. The diagnosis model was evaluated by ROC analysis (Fig. S1), which revealed an area under the curve (AUC) of 0.9977 and specificity and sensitivity of 98.55% and 99.32%, respectively. Meanwhile, the AUC for ARG was 0.7026, with a specificity of 50.72% and a sensitivity of 83.56%. The AUC of C0 was 0.7347, with specificity and sensitivity of 85.51% and 59.59%, respectively, and the AUC of C6 was 0.8791 (specificity of 79.71% and sensitivity of 83.56%). The AUC of C6DC was 0.9896, with a specificity of 94.2% and a sensitivity of 97.95%, while C16OH had an AUC of 0.9772, with a specificity of 97.1% and a sensitivity of 89.04%. Therefore, the combination of ARG, C0, C6, C16OH and C6DC is more effective in discriminating gastric cancer from atrophic gastritis and superficial gastritis (Table 2).

**Table 1:** Patient features in the SG, AG and GC groups.

	SG	AG	GC
N	72	74	69
Age (*Median* (*interquartile range*))	49 (29)	50 (16.25)	62 (14)^*^^#^
Sex (male/female)	18/54	45/29^a^	47/22^#^
*Pylori* antibodies (+/-)	20/52	30/44	31/38^#^
**TNM stage**			
I	NA	NA	9
II	NA	NA	37
III	NA	NA	20
IV	NA	NA	3
**Tumor location**			
Gastric antrum	NA	NA	32
Gastric body	NA	NA	24
Whole gastric	NA	NA	6
fundus-cardia	NA	NA	7
**Tumor differentiation**			
poor	NA	NA	39
moderate	NA	NA	23
high	NA	NA	7

**Notes.**

* *P* < 0.05, GC versus AG.

# *P* < 0.05, GC versus SG.

a *P* < 0.05, AG compared with SG.

## Discussion

Gastric cancer (GC) is among the most common cancers worldwide, which is considered a multistage progressive process (Kuligowski et al., 2016). AG is currently assessed as a pre-malignant gastric lesion in patients at risk of progression to GC, as expected in the Correa’s cascade model, which is a widely accepted GC pathogenesis model (Annibale, Esposito & Lahner, 2020). New noninvasive biomarkers of GC has become an active field of research that would improve the detection rate of early GC and therefore the prognosis of this prevalent neoplasia.

In this study, we investigated the 42 metabolites variation using serum from patients with GC, AG and SG. Our experimental found changes in serum metabolomics during the whole process from SG to AG and then to GC, and constructed a panel of metabolomic markers including C6DC, C16OH, C6, C0 and ARG that was suitable for the diagnosis of GC. Moreover, our results showed that the diagnosis model sensitivity and specificity in GC diagnosis were 98.55% and 99.32%.

As shown above, GC cases had elevated plasma ARG levels compared with SG and AG patients, in agreement with a study examining gastric juices amples and a study examining plasma ARG (Liu et al., 2018; Shi et al., 2021), but in contrast with Jing et al. (2018). ARG contributes to the metabolism of nitric oxide (NO), a vasodilator and free radical involved in inflammatory reactions and cancer development *via* nitro-oxidative stress, apoptosis, cell cycle, angiogenesis, invasion and metastasis (Ni et al., 2019). High ARG amounts were considered to be responsible for NO elevation (Yang, Taboada & Liao, 2009) Inducible nitric oxide synthase (iNOS) was suggested to promote NO production in GC initiation and progression (Yang, Taboada & Liao, 2009). Moreover, high levels of argininosuccinate synthase 1 (ASS1) induce tumor growth and aggressiveness by elevating ARG amounts for NO synthesis (Keshet & Erez, 2018). High NO flux leads to the formation of reactive nitrogen species (RNS) that causes deoxyribonucleic acid (DNA) damage and/or mutations, which could eventually induce carcinogenesis in gastric cancer (Wink et al., 1991). Also, the NO flux can induce cMyc mutation through the EGFR pathway (Somasundaram et al., 2019) (Fig. S2). Results of this study demonstrated that alanine, citrulline, leucine, methionine, phenylalanine, proline, tyrosine, valine, glycine and ornithine could not distinguish GC from AG and SG. This result differed slightly from those of previous studies. Miyagi et al. (2011) reported threonine, glutamine, alanine, citrulline, leucine, phenylalanine, lysine, asparagine, methionine, citrulline, valine, tryptophan, histidine and ARG amounts are reduced in GC patients, ornithine, proline, glycine levels were not notably different between GC and healthy group (Miyagi et al., 2011). Jing et al. (2018) studied the amino acid profiles of gastric cancer and gastric ulcers patients, and found glutamine, histidine, ARG and tryptophan reduced amounts, whereas ornithine content was increased in plasma specimens from patients with GC. They furtherly established regression model showed AUC at 0.922 with 85.5% specificity and 89.1% sensitivity. The most likely reason for differences between our findings and previously published studies is the different criteria for sample grouping.

**Table 2:** ROC curve analysis of diagnostic indicators in differentiating GC from AG and SG.

	**Regressionmodel**	**C16OH**	**C6DC**	**C6**	**C0**	**ARG**
**GCvsSG**						
AUC	0.9986	0.9779	0.9910	0.8793	0.7385	0.6818
Cut-off value		0.0555	0.227	0.0545	11.29	37.25
Sensitivity (%)	98.55	90.28	97.22	76.39	65.28	83.33
Specificity (%)	98.55	97.1	95.65	89.86	81.16	50.72
*P* value	<0.0001	<0.0001	<0.0001	<0.0001	<0.0001	0.0002
**GCvsAG**						
AUC	0.9961	0.9765	0.9882	0.8789	0.7309	0.7229
Cut-off value		0.0555	0.237	0.0625	9.462	30.99
Sensitivity (%)	98.55	87.84	98.65	82.43	54.05	75.68
Specificity (%)	100	97.1	94.2	79.71	91.3	63.77
*P* value	<0.0001	<0.0001	<0.0001	<0.0001	<0.0001	<0.0001
**GCvs(AG+SG)**						
AUC	0.9977	0.9772	0.9896	0.8791	0.7347	0.7026
Cut-off value		0.0555	0.237	0.0625	10.59	37.25
Sensitivity (%)	99.32	89.04	97.95	83.56	59.59	83.56
Specificity (%)	98.55	97.1	94.2	79.71	85.51	50.72
*P* value	<0.0001	<0.0001	<0.0001	<0.0001	<0.0001	<0.0001

Acylcarnitines enter the mitochondria, where they are converted by *CPT2* into their CoA esters, which then undergo *β*-oxidation (Houten & Wanders, 2010). As such event is important in fatty acid metabolism and intracellular CoA homeostasis, acylcarnitine accumulation is associated with insufficient fatty acid oxidation and reduced mitochondrial function (Adeva-Andany et al., 2019) (Fig. S3). Intracellular acylcarnitine amounts might reflect serum levels (McGill et al., 2014). Here, we found that C6DC, C16OH, C6 and C0 amounts were elevated in GC cases. Upregulation of the *β*-oxidation process might increase C6DC, C16OH, C6 and C0 amounts. Therefore, reducing in C6DC, C16OH, C6 and C0 concentrations maybe a novel approach for cancer therapy. Corona et al. (2018) reported GC patients are characterized by increased amounts of acylcarnitine derivatives (C2, C16 and C18:1), corroborating the current findings.

Furthermore, ROC curve was built in the present study to evaluate the diagnostic values of the semetabolites. The combined detection factor had a higher area under the ROC curve compared with each single metabolite. The AUC of the combined detection factor was 0.9977, with specificity and sensitivity of 98.55% and 99.32%, respectively. This indicated that the diagnostic performance of the combined metabolites was obviously higher than that of each single metabolite. Moreover, the combined diagnostic panel had a favorable diagnostic effect, providing a new basis for the early diagnosis of GC. However, this was a single-center trial including few patients. Future trials with larger patient cohorts are needed to substantiate the above results.

## Conclusion

In summary, increased C6DC, C16OH, C6, C0 and ARG expression distinguished GC from AG and SG, indicating C6DC, C16OH, C6, C0 and ARG are potential diagnostic markers of GC. This study was based on observed metabolite changes during the whole process from SG to AG and then to GC. Thus, our study provides insights in understanding metabolite profiles of amino acids and acylcarnitines also associated with gastric cancer and their use as cancer biomarkers. Further studies with larger patient cohorts to further assess clinical relevance are needed as patients is still limited.

##  Supplemental Information

10.7717/peerj.14115/supp-1 Supplemental Information 1 STROBE checklist

10.7717/peerj.14115/supp-2 Supplemental Information 2 Supplementary figures

10.7717/peerj.14115/supp-3 Supplemental Information 3 Data and statistics

## Abbreviations

GC: gastric cancer
AG: atrophic gastritis
SG: superficial gastritis
cfNAs: Cell-free nucleic acids
NMR: nuclear magnetic resonance spectroscopy
MS: mass spectroscopy
LC: liquid chromatography
LC-MS/MS: LC-tandem mass spectroscopy
DSS: dried serum spot
VIP: Variable importance inprojection
OPLS-DA: orthogonal partial least squares discriminant analysis
ROC: Receiver operating characteristic
C0: free carnitine
C2: acetylcarnitine
C3: propionylcarnitine
C3DC: malonylcarnitine
C4OH: 3-hydroxy-butyrylcarnitine
C4: butyrylcarnitine
C4DC: methylmalonyl
C5OH: 3-hydroxy-isovalerylcarnitine
C5: isovalerylcarnitine
C5:1: tiglylcarnitine
C5DC: glutarylcarnitine
C6OH: 3-hydroxy-hexanoylcarnitine
C6: hexanoylcarnitine
C6DC: adipylcarnitine
C8: octanoylcarnitine
C8:1: octenoylcarnitine
C10: decanoylcarnitine
C10:1: decanoylcarnitine
C10:2: decadienoylcarnitine
C12: dodecanoylcarnitine
C12:1: dodecanoylcarnitine
C14: tetradecanoylcarnitine
C14:1: tetradecenoyl-carnitine
C14OH: 3-hydroxy-tetradecanoylcarnitine
C14:2: tetradecadienoylcarnitine
C16: hexadecanoylcarnitine
C16OH: 3-hydroxy-hexadecanoylcarnitine
C16:1: hexadecenoylcarnitin
C16:1OH: 3-hydroxy-hexadecenoylcarnitine
C18: octadecanoylcarnitine
C18OH: 3-hydroxy-octadecanoylcarnitine
C18:1: octadecenoylcarnitine
C18:1OH: 3-hydroxy-octadecenoylcarnitine
C18:2: octadecadienoylcarnitine
NO: nitric oxide
iNOS: Inducible nitric oxide synthase
RNS: reactive nitrogen species
DNA: deoxyribonucleic acid

## Additional Information and Declarations

### Data Availability

The following information was supplied regarding data availability: The raw data is available in the Supplemental Files.
