# Supplementary material for: Targeted metabolomic profiles of serum amino acids and acylcarnitines related to gastric cancer
Source: PeerJ. 2022 Oct 6;10:e14115. doi: 10.7717/peerj.14115 (PMC9548315; doi:10.7717/peerj.14115)
Supplement: Supplemental Information 2 [file peerj-10-14115-s002.docx]

Targeted Metabolomic Profiles of Serum Amino Acids and Acylcarnitines Related to Gastric Cancer

Running title: Metabolomic Profiles in Gastric Cancer

Dehong Li^1,2^, Yan Lu^2^, Fenghui Zhao^3^, Li Yan^2^, Xingwen Yang^2^, Lianhua Wei^2^, Xiaoyan Yang^2^, Xiumei Yuan^2^, Kehu Yang^1*^

^1^Evidence Based Medicine Center, School of Basic Medical Sciences, Lanzhou University, Lanzhou 730000, China

^2^Department of Clinical laboratory, Gansu Provincial Hospital, Lanzhou 730000, China

^3^Department of Pathology, Gansu Provincial Hospital, Lanzhou 730000, China

^*^Correspondence:

Kehu Yang

Evidence Based Medicine Center, School of Basic Medical Sciences,Lanzhou University, Lanzhou, 730000, China, Tel: +86-13893117077; Email: [yangkh-ebm@lzu.edu.cn](mailto:yangkh-ebm@lzu.edu.cn)


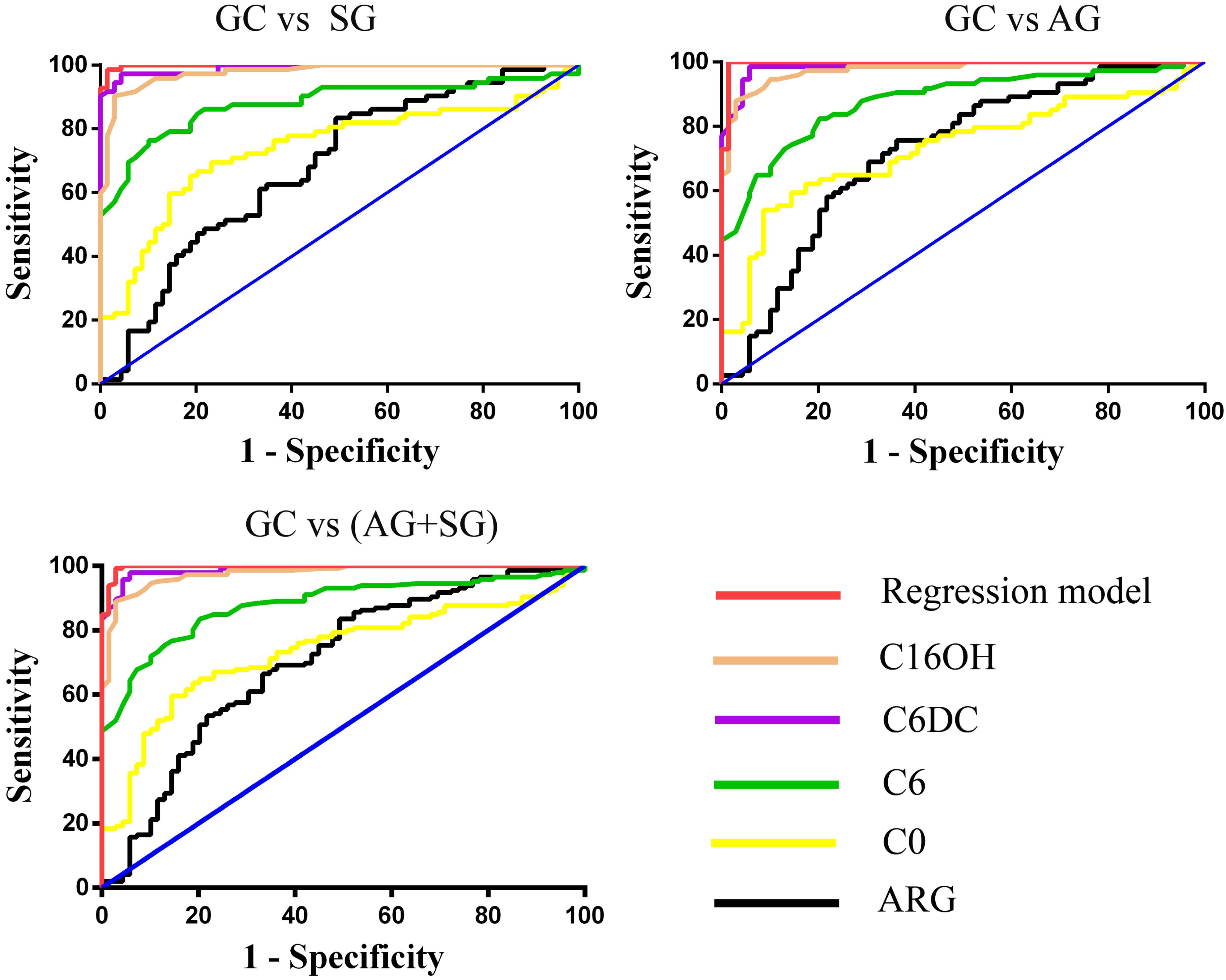


**Figure.S1.**Receiver operating characteristic (ROC) curves for five metabolites and the regression model. AUC, area under the curve.


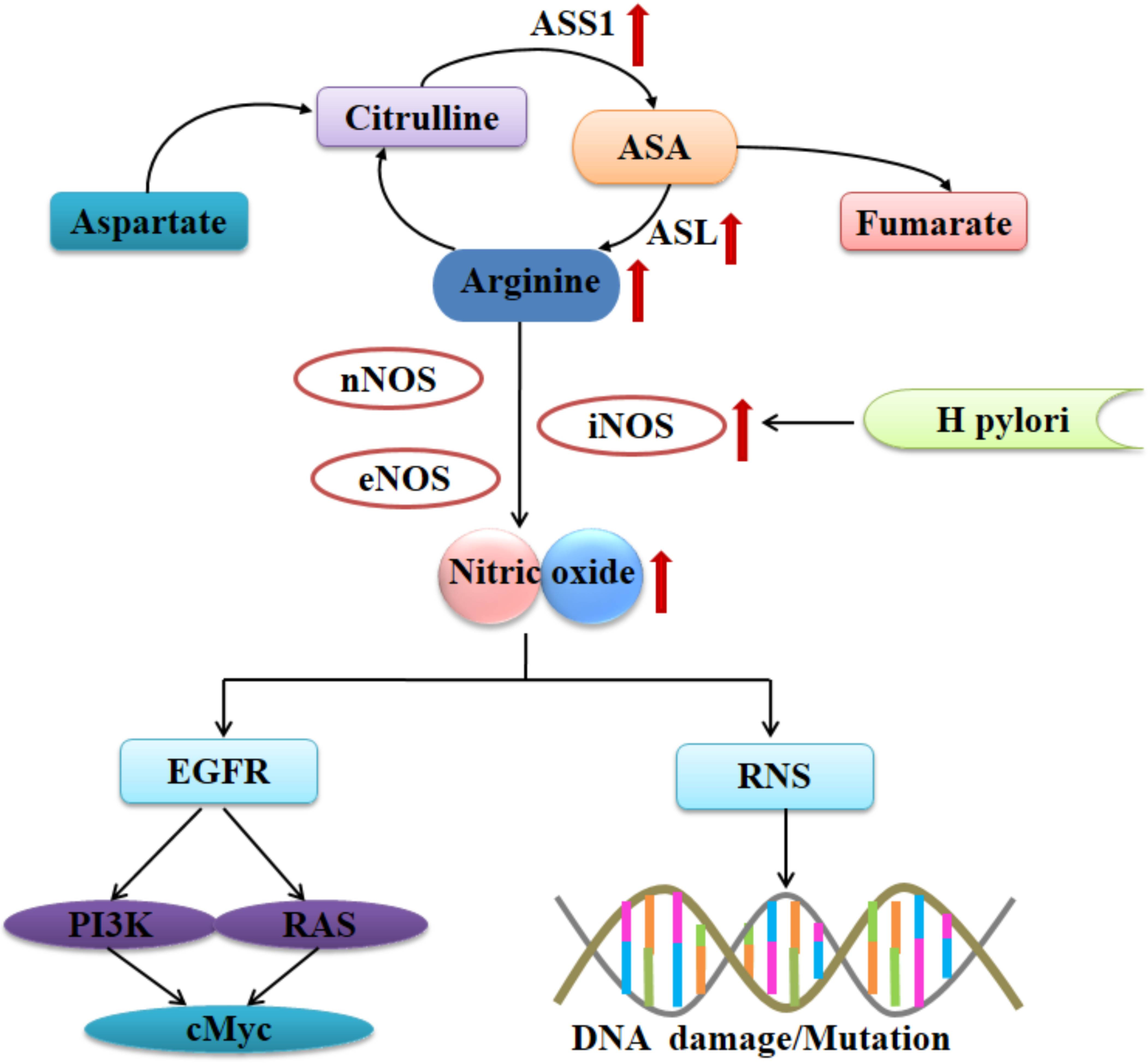


**Figure.S2.Molecular mechanisms of arginine involvement in gastric cancer**. Red arrows denote cancer-related upregulation of proteins contributing to arginine metabolism, causing a net increase in nitric oxide (NO) synthesis. Cancer cells showed enhanced NO amounts by upregulating iNOS, ASS1 and ASL, which increase arginine availability for nitric oxide synthesis.

ASA, argininosuccinic acid; ASL,argininosuccinatelyase; ASS1, argininosuccinate synthase 1; NOS:nitricoxide synthase;RNS:reactive nitrogen species


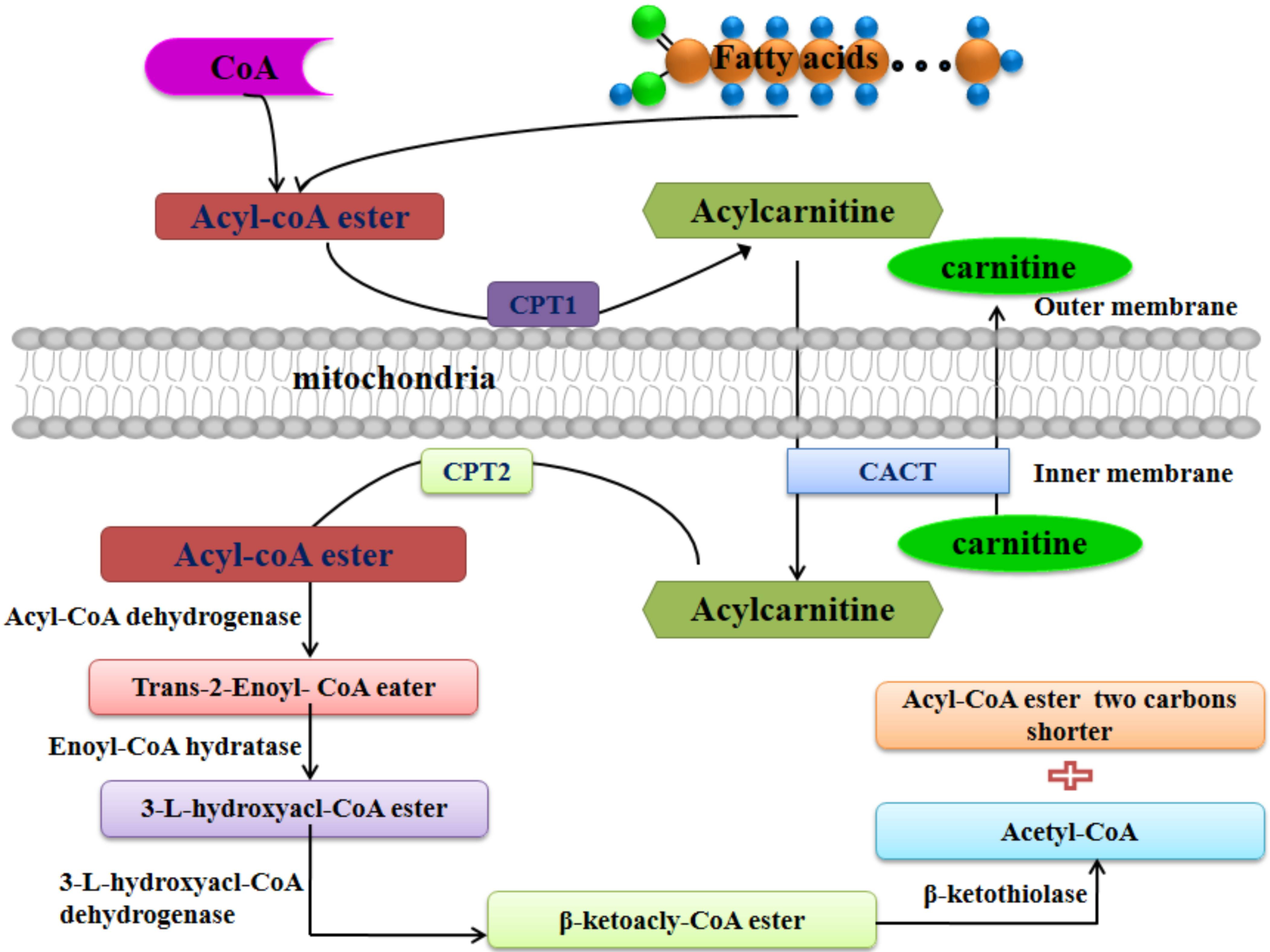


**Figure.S3.Mitochondrial fatty acid β-oxidation.** Upon transport across the cytosolic membrane, fatty acids are transformed into acyl-CoAs in the cytosol. CPT1 transforms acyl-CoAs into respective acylcarnitines, which are then transported across the mitochondrial membrane by CACT. CPT2 converts the seacylcarnitines back into acyl-CoAs, which are degraded into acetyl-CoAs via an inducible set of enzymes.

CPT:carnitinepalmitoyltransferase; CACT: carnitine-acylcarnitinetranslocase
